# A crossover comparison of patient satisfaction with two teriparatide regimens: primary results of the Japanese Osteoporosis Intervention Trial 06 (JOINT-06)

**DOI:** 10.1007/s00774-024-01521-7

**Published:** 2024-06-11

**Authors:** Satoshi Soen, Yukari Uemura, Shiro Tanaka, Yasuhiro Takeuchi, Naoto Endo, Junichi Takada, Satoshi Ikeda, Jun Iwamoto, Nobukazu Okimoto, Sakae Tanaka

**Affiliations:** 1 Soen Orthopaedics, Osteoporosis and Rheumatology Clinic, Kobe, Hyogo Japan; 2 Biostatistics Section, Department of Data Science, Center for Clinical Sciences, National Center for Global Health and Medicine, Tokyo, Japan; 3 Department of Clinical Biostatistics, Graduate School of Medicine, Kyoto University, Kyoto, Japan; 4 Toranomon Hospital Endocrine Center, Okinaka Memorial Medical Research, Minato-Ku, Tokyo, Japan; 5 Department of Orthopedic Surgery, Saiseikai Niigata Kenoh Kikan Hospital, Sanjo City, Niigata Japan; 6 Osteoporosis Center, Sapporo Maruyama Orthopaedic Hospital, Sapporo, Hokkaido Japan; 7 Department of Orthopaedic Surgery, Ken-Ai Memorial Hospital, Onga, Fukuoka Japan; 8 Bone and Joint Disease Center, Keiyu Orthopaedic Hospital, Gunma, Tatebayashi, Japan; 9 Okimoto Clinic, Kure, Hiroshima Japan; 10 Department of Orthopaedic Surgery, Faculty of Medicine, The University of Tokyo, Tokyo, Japan

**Keywords:** Osteoporosis, Once-daily vs twice-weekly self-injection, Patient satisfaction, Randomized controlled trial, Teriparatide

## Abstract

**Introduction:**

This study aimed to compare treatment satisfaction with two dosing regimens (two teriparatide [TPTD] self-injection systems) in osteoporosis patients at high risk of fracture.

**Materials and methods:**

In this open-label crossover randomized trial comparing self-injected once-daily (1/D)-TPTD with self-injected twice-weekly (2/W)-TPTD, three satisfaction variables were evaluated by questionnaire for 2 years. The primary endpoint was overall satisfaction and secondary endpoints were satisfaction with treatment effectiveness and with utility of the self-injection device. Changes in quality of life (QOL) assessed by EuroQol-5 Dimension, pain assessed by visual analogue scale (VAS), and anthropometric parameters were also analyzed. Safety was evaluated based on the incidence and severity of adverse events (AEs).

**Results:**

The 1/D-TPTD and 2/W-TPTD groups consisted of 180 (75.9 ± 7.3 years) and 179 (age: 75.5 ± 6.9 years) patients, respectively. After 26 weeks of treatment, no significant between-group difference in the persistence rate (79.4% vs 72.6% in the 1/D-TPTD and 2/W-TPTD groups, respectively), distributions of overall satisfaction scores, and satisfaction with treatment (*p* > 0.05) were observed. However, several items of satisfaction with the utility of the injection device were significantly higher in the 2/W-TPTD group (*p* < 0.05). Statistical improvements from baseline values were observed in QOL and pain VAS in both groups (*p* < 0.05). No serious AEs were reported.

**Conclusion:**

The between-group similarity of overall treatment satisfaction and effectiveness scores and between-group difference in satisfaction with the utility of the self-injection device was useful information for real-world treatment of osteoporosis. Both medication regimens were well tolerated.

**Supplementary Information:**

The online version contains supplementary material available at 10.1007/s00774-024-01521-7.

## Introduction

Osteoporosis is a chronic disorder characterized by low bone mass and disordered skeletal microarchitecture, resulting in impaired bone strength and an increased risk of fragility fracture [1].

Several pharmacological agents are available to lower fracture risk, either by reducing bone resorption or by stimulating bone formation [2]. Recent studies suggest that bone anabolic agents have important roles in the initial treatment of patients with osteoporosis, especially for those at very high risk of fracture [3]. Moreover, it was reported that bone mineral density (BMD) accrual is maximized when patients are given anabolic agents first, followed by potent antiresorptive therapy [4].

Nonadherence to pharmacological agents in osteoporosis is a well-recognized problem. Treatment discontinuation due to poor adherence places an enormous burden on patients by increasing rates of fractures and use of healthcare resources [5, 6]. Actually, a meta-analysis of existing studies reported that good adherence, compared with nonadherence, significantly reduced the risk of all fractures by 28%, the risk of hip fracture by 49%, and the risk of non-vertebral fracture by 26% [7].

Extending the dosing interval of bone resorption inhibitor improves medication adherence [8]. The extension of dosing intervals may be one element contributing to improvement in therapeutic adherence. Other elements may be improved patient education, enhanced healthcare provider–patient interaction, taking into account patient's preferences, and involving them in treatment decisions [5].

In Japan, the once-daily [9], once-weekly [10], and twice-weekly [11] teriparatide dosing regimens have similar efficacy and have been approved for the clinical treatment of high-risk osteoporosis. Two of these regimens, the once-daily and twice-weekly, involve the use of self-injectable formulations and are widely used. The self-injection device used to deliver the twice-weekly formulation has an invisible needle that does not require replacement, and contains a much lower total dose of teriparatide than used in the once-daily formulation. These benefits are expected to lower the number of needle stick accidents and reduce self-administration complexity [12, 13], thereby improving patients’ satisfaction and adherence.

Although patient satisfaction with the osteoporosis antiresorptive agents, denosumab and bisphosphonates, has been studied in a crossover manner [14, 15], differences in treatment satisfaction between formulations of teriparatide have not been compared in a similar manner.

The primary objective of this study was to compare once-daily and twice-weekly self-injectable formulations of teriparatide by evaluating patient satisfaction (including both adherence and persistence), efficacy, and safety in a prospective, open-label, crossover, randomized trial. This report describes the results from the first 26 weeks, which includes the primary endpoint of the study.

## Materials and methods

### Ethical approval

All procedures performed in studies involving human participants were in accordance with institutional and/or national research committee ethical standards and with the ethical principles set out in the Declaration of Helsinki and its amendments or comparable ethical standards. The study was also conducted in compliance with the Clinical Trials Act and related ministerial orders, and all applicable regulations and ethical requirements. The study protocol was approved by the Toranomon Hospital Certified Review Board. Written informed consent was obtained from all individual participants prior to inclusion in the study.

### Study subjects

This multicenter open-label crossover study (jRCTs031210187) planned to enroll 400 postmenopausal women with primary osteoporosis [16], aged 60 years or older. Patients at high fracture risk were eligible if they satisfied any of the following inclusion criteria: BMD < 60% of the young adult mean (YAM) or < − 3.3 standard deviation (SD); ≥ 2 vertebral fractures (assessed by a semi-quantitative method [17]) between the fourth thoracic vertebra (Th4) and fourth lumbar vertebra (L4); a grade 3 vertebral fracture; ≥ 1 spinal vertebral fracture at Th4–L4 and BMD ≤ -2.5 SD of YAM; or history of hip fracture. Patients were excluded if they had (1) a diagnosis of secondary osteoporosis; (2) bone loss induced by diseases other than osteoporosis; (3) hypersensitivity; (4) any contraindication to teriparatide; (5) severe hepatic failure, renal failure, or cardiac failure; (6) inability to self-administer the drug; (7) experience with using any auto-injection device; (8) dementia when medical interview of the patient was difficult to conduct; (9) receipt of an investigational trial anti-osteoporosis drug within the 52 weeks prior to giving informed consent; (10) been hospitalized; (11) a history of teriparatide treatment.

### Study design

After providing their informed, written consent to participate, eligible subjects were randomly divided into two groups. One group received a once-daily dose (20 μg self-injection) of teriparatide (1/D-TPTD group) and the other, a twice-weekly dose (28.2 μg self-injection) of teriparatide (2/W-TPTD group) for 26 weeks (the first quarter of the 104-week study period). The dosing regimen was then switched and treatment was continued for another 26 weeks (one half of the total study period). After completion of the 52-week crossover study, patients were allowed to receive either a daily dose or twice-weekly dosing depending on their preference, and the treatment continued for another 52 weeks (ending the study). All patients received daily vitamin D supplementation (25 μg/day) throughout the study period.

There was no basis for setting the number of cases to statistically test the primary outcome hypothesis. Therefore, in consultation with other researchers, we determined that a group difference of at least 1 point would be a clinically meaningful and large difference, and assumed a group difference of 0.7 to 1.0. We further assumed a score variability of SD 1.4 to 2.4 and set the number of cases per group at 200.

### Endpoints

Patient satisfaction was investigated using the Patient Satisfaction Questionnaire (Online Resource 1). The questionnaire consists of one question on overall satisfaction (asked at 26 weeks), two questions on the effectiveness of treatment (asked at 26 weeks), and 12 questions on the utility of self-injection device (asked at 2, 4, 13, and 26 weeks) with ease of use rated from difficult to easy on a 3- or 6-point scale. The primary endpoint of the study was the degree of overall patient satisfaction at 26 weeks. The secondary endpoints were (1) overall patient satisfaction at 52 and 104 weeks, (2) patient satisfaction with treatment at 26, 52, and 104 weeks, (3) time course of patient satisfaction with the device utility (12 questions), (4) preference when the patient was allowed to choose at 52 weeks, (5) adherence, and (6) efficacy of the treatment. Adherence was judged to be good if self-injection rate was ≥ 80%, self-injection rate was ≥ 50% during the final 4 weeks, and attendance at a final visit was within the predetermined time limit. Treatment efficacy was judged by the number of incident vertebral or non-vertebral clinical fractures, change of BMD, quality of life (QOL) on the EuroQol-5 Dimension (EQ-5D) scale, pain evaluated on a visual analogue scale (VAS), and parameters of bone structure analysis. Clinical fractures were identified through monitoring clinical symptoms and confirmed with radiography by the physicians. BMDs were measured at L1–L4 or L2–L4, the femoral neck, and total hip by dual energy X-ray absorptiometry. Safety of treatment was evaluated based on incidence, type, and severity of adverse events (AEs). AEs were evaluated by the attending physician through an interview when the patient visited the institute.

### Statistical analyses

Endpoints were analyzed in the full analysis set (FAS). All data are presented as mean ± SD or number and percentage. The differences in baseline characteristics between the 1/D-TPTD and 2/W-TPTD groups were evaluated by the *t*-test and Fisher χ^2^ test used for continuous variables and categorical variables, respectively. The difference between the average score for the primary endpoint was compared using the *t* test. Also, the differences in score distribution between groups were evaluated using the Cochran-Mantel–Haenszel test. Other patient satisfaction endpoints were also analyzed as continuous variables using the *t* test. The paired *t* test was used to assess time-dependent changes in QOL, VAS, and anthropometric parameters from baseline. Also, incidences of clinical fractures were compared using the Fisher exact test. Differences with *p* values less than 0.05 were considered statistically significant. All statistical analyses were performed using SAS software version 9.4 (SAS Institute, Cary, NC).

## Results

A total of 381 patients (190 in the 1/D-TPTD group and 191 in the 2/W-TPTD group) were enrolled between July 2021 and September 2022. Twenty patients were excluded from analysis mainly due to canceled participation prior to the start of treatment and the remaining 359 patients, 180 and 179 for the 1/D-TPTD and 2/W-TPTD group, respectively, served as the FAS population.

### Patient characteristics

Table 1 summarizes patient characteristics. The mean and standard deviation of age at the time of registration was 75.9 ± 7.3 years in the 1/D-TPTD group and 75.5 ± 6.9 years in the 2/W-TPTD group. Approximately 31% of patients in each group had a clinical fracture within 1 month before randomization. No significant differences in those listed characteristics were observed between the two groups (*p* > 0.05). The most common anti-osteoporosis drugs used prior to the start of TPTD were bisphosphonates (18.4%), followed by eldecalcitol (16.4%).

**Table 1:** Baseline characteristics of patients

Items	1/D-TPTD (*N* = 180)	2/W-TPTD (*N* = 179)	Overall (*N* = 359)	*p*
Age, years old	75.9 (7.3)	75.5 (6.9)	75.7 (7.1)	0.568
Age at menopause, years old	50.1 (4.8)	49.8 (3.3)	50.0 (4.1)	0.578
Height, cm	150.3 (6.1)	150.0 (6.1)	150.2 (6.1)	0.660
Body weight, kg	49.6 (7.7)	49.9 (8.8)	49.8 (8.2)	0.677
BMI, kg/m^2^	22.0 (3.2)	22.2 (3.7)	22.1 (3.4)	0.566
BMD				
Lumber spine (L2–L4), T-score	− 2.27 (1.8)	− 2.48 (1.3)	− 2.38 (1.6)	0.276
Femoral neck, T-score	− 3.29 (0.8)	− 3.22 (0.9)	− 3.26 (0.9)	0.441
Total hip, T-score	− 2.54 (0.9)	− 2.58 (1.0)	− 2.56 (1.0)	0.765
EQ-5D, utility score	0.88 (0.3)	0.91 (0.2)	0.90 (0.2)	0.192
VAS	36.0 (69.8)	28.5 (29.5)	32.2 (54.0)	0.190
Clinical fracture within 1 month				
Yes	56 (31.1%)	57 (31.8%)	113 (31.5%)	0.910
No	124 (68.9%)	122 (68.2%)	246 (68.5%)	
History of hip fracture				
Yes	15 (8.3%)	16 (8.9%)	31 (8.6%)	0.853
No	165 (91.7%)	163 (91.1%)	328 (91.4%)	
Complications				
Yes	99 (55.0%)	93 (52.0%)	192 (53.5%)	0.597
No	81 (45.0%)	86 (48.0%)	167 (46.5%)	
Hyperlipidemia	40 (22.2%)	42 (23.5%)	82 (22.8%)	
Hypertension	80 (44.4%)	73 (40.8%)	153 (42.6%)	
Diabetes	18 (10.0%)	10 (5.6%)	28 (7.8%)	
Chronic nephrites	2 (1.1%)	2 (1.1%)	4 (1.1%)	
Previous treatment for osteoporosis				
Yes	65 (36.1%)	80 (44.7%)	145 (40.4%)	0.107
No	115 (63.9%)	99 (55.3%)	214 (59.6%)	

Values are indicated as the mean and standard deviation or number and percentage. Differences between groups were evaluated by the *t* test or Fisher *χ*^2^ test

*1/D-TPTD* once-daily dose of teriparatide, *2/W-TPTD* twice-weekly dose of teriparatide, *BMI* body mass index, *BMD* bone mineral density, *EQ-5D* EuroQol-5 Dimension, *VAS* visual analogue scale

### Persistence

During the 26-week observation period, 37 patients in the 1/D-TPTD group and 49 patients in the 2/W-TPTD group stopped the treatment, and thus the continuation rate at the end of 26 weeks was 79.4% and 72.6%, respectively (*p* = 0.671).

### Endpoints

The degree of overall patient satisfaction, the primary endpoint of the study, is summarized in Table 2. The distributions of overall satisfaction scores in both groups were similar and no significant difference was observed between the groups (*p* = 0.522). The overall satisfaction score, treated as a continuous variable, was 3.5 ± 1.3 in the 1/D-TPTD group and 3.4 ± 1.2 in the 2/W-TPTD group and not significantly different between the groups (*p* = 0.523). The scores of patient satisfaction with the effectiveness of treatment at 26 weeks were also similar in both groups, and no significant difference was observed (*p* > 0.05, Table 2).

**Table 2:** Patient overall satisfaction and satisfaction with treatment

Items				Response (*N*, %)						
				0	1	2	3	4	5	*p*
Overall satisfaction			1/D-TPTD	6 (3.3%)	6 (3.3%)	11 (6.1%)	50 (27.8%)	36 (20.0%)	39 (21.7%)	0.522
			2/W-TPTD	2 (1.1%)	7 (3.9%)	19 (10.6%)	50 (27.9%)	23 (12.8%)	35 (19.6%)	
Satisfaction with treatment	Q1	On the teriparatide preparation in use, do you feel positive effects? Please rate the effects on a scale of 0 to 5	1/D-TPTD	27 (15.0%)	16 (8.9%)	11 (6.1%)	39 (21.7%)	32 (17.8%)	23 (12.8%)	0.342
			2/W-TPTD	28 (15.6%)	17 (9.5%)	19 (10.6%)	31 (17.3%)	14 (7.8%)	27 (15.1%)	
	Q2	On the teriparatide preparation in use, do you feel improvement of lower or upper back pain? Please rate the improvement on a scale of 0 to 5	1/D-TPTD	28 (15.6%)	14 (7.8%)	11 (6.1%)	36 (20.0%)	31 (17.2%)	28 (15.6%)	0.091
			2/W-TPTD	32 (17.9%)	14 (7.8%)	16 (8.9%)	35 (19.6%)	19 (10.6%)	20 (11.2%)	

Values are indicated as number and percentage. Differences between groups were tested by using the Cochran-Mantel–Haenszel-test

*1/D-TPTD* once-daily dose of teriparatide, *2/W-TPTD* twice-weekly dose of teriparatide

**Fig. 1:**
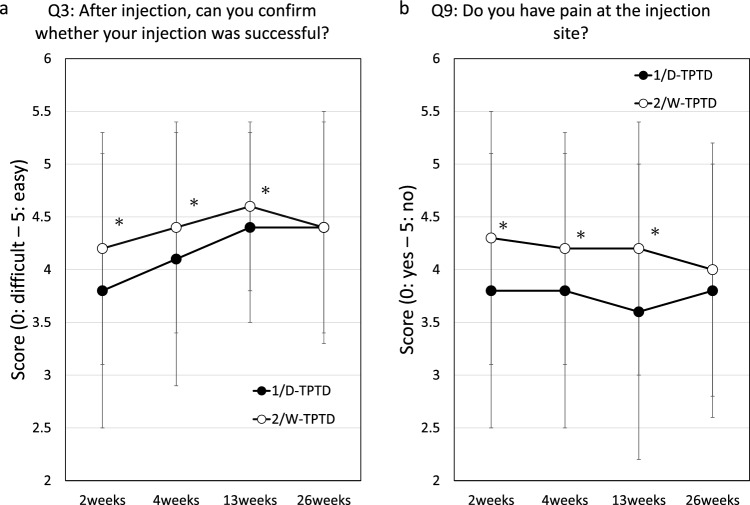
Time course of the rate of patient satisfaction with injection device utility corresponding to **a** Q3 and **b** Q9. Each point and bar indicate the mean score ± standard deviation. Asterisk indicates *p* < 0.05 between the groups. *1/D-TPTD* once-daily dose of teriparatide, *2/W-TPTD* twice-weekly dose of teriparatide

Scores of patient satisfaction with the utility of the injection device (12 questions each) and the overall scores are summarized in Online Resource 2. The results show significantly higher patient satisfaction score (the sum of nine item scores corresponding to Q1, Q2, Q3, Q5, Q6, Q7, Q8, Q9, and Q12) in the 2/W-TPTD group at 2 weeks after the start of treatment (*p* < 0.05) but no difference in the sums of two item scores corresponding to Q10 and Q11 at any time point. Scores corresponding to Q3 (Confirmation that the injection was successful) and Q9 (Pain at the injection site) were also higher in the 2/W-TPTD group at 4 and 13 weeks, and the score corresponding to Q4 (Have you failed to inject the medication?) was lower at 4 and 26 weeks. The time course of the mean values of those two scores (Q3, 9) is shown in Fig. 1a and b.

Clinical fracture occurred in 4 patients in the 2/W-TPTD group (1 vertebral fracture and 3 non-vertebral fractures), but not the 1/D-TPTD group. However, the difference between the groups was not statistically significant (*p* = 0.061). QOL at 26 weeks was significantly improved from the baseline value in both groups (Table 3). The heights of patients significantly decreased at 26 weeks in the both groups, whereas body weight was significantly decreased in the 2/W-TPTD group (*p* = 0.001). AEs occurred in 10 patients (5.6%) in the 1/D-TPTD group and 13 patients (7.2%) in the 2/W-TPTD group, none of which were serious (Table 4).

**Table 3:** Effects on quality of life, pain visual analogue scale, body height, and weight

		1/D-TPTD			2/W-TPTD			
		Mean (SD)	*N*	p*	Mean (SD)	*N*	p*	p**
EQ-5D, utility score	Baseline	0.88 (0.3)	180		0.91 (0.2)	177		
	26 weeks	0.95 (0.2)	152		0.98 (0.1)	138		
	Absolute change	0.07 (0.2)	152	< 0.001	0.05 (0.2)	137	0.010	0.961
VAS	Baseline	36.0 (69.8)	179		28.5 (29.5)	175		
	26 weeks	20.6 (22.5)	149		22.0 (25.5)	136		
	Absolute change	− 14.7 (75.4)	148	0.019	− 6.0 (32.4)	134	0.035	0.162
Height, cm	Baseline	150.3 (6.1)	171		150.0 (6.1)	172		
	26 weeks	149.6 (6.4)	128		150.2 (6.3)	117		
	Absolute change	− 0.5 (1.8)	127	0.002	− 0.3 (1.4)	114	0.023	0.126
Body weight, kg	Baseline	49.56 (7.7)	171		49.9 (8.8)	172		
	26 weeks	49.65 (8.0)	129		50.2 (8.9)	118		
	Absolute change	− 0.33 (2.4)	128	0.128	− 0.7 (2.3)	115	0.001	0.661

Changes of parameters were evaluated by paired *t* test (*). Differences between groups were tested using the *t* test (**)

*1/D-TPTD* once-daily dose of teriparatide, *2/W-TPTD* twice-weekly dose of teriparatide, *SD* standard deviation, *EQ-5D* EuroQol-5 Dimension, *VAS* visual analogue scale

**Table 4:** Adverse events reported during the study period

	1/D-TPTD (N = 180)		2/W-TPTD (N = 181)	
	10 (5.6%)		13 (7.2%)	
	Serious	Not serious	Serious	Not serious
Total AEs		11		22
Chills	0	0	0	1 (0.6%)
Nausea	0	4 (2.2%)	0	6 (3.3%)
Gastrointestinal disorders	0	0	0	1 (0.6%)
Diarrhea	0	2 (1.1%)	0	1 (0.6%)
Arthralgia	0	0	0	1 (0.6%)
Hypercalcemia	0	1 (0.6%)	0	0
Decreased appetite	0	1 (0.6%)	0	0
Spinal compression fracture	0	1 (0.6%)	0	0
Headache	0	0	0	3 (1.7%)
Palpitation	0	0	0	1 (0.6%)
Discomfort	0	0	0	2 (1.1%)
Dizziness	0	0	0	1 (0.6%)
Abdominal pain	0	0	0	1 (0.6%)
Drug eruption	0	2 (1.1%)	0	0
Vomiting	0	0	0	2 (1.1%)

Values are indicated as a nubmber of patients and percentage

*1/D-TPTD* once-daily dose of teriparatide, *2/W-TPTD* twice-weekly dose of teriparatide, *AE* Adverse event

## Discussion

This is a preliminary report on our ongoing comparative crossover study to evaluate patient satisfaction with two types of teriparatide self-injection systems, the 1/D-TPTD and 2/W-TPTD. The results of evaluation conducted at 26 weeks after the start of treatment, that is, just before the crossover, demonstrated that the degree of overall patient satisfaction for both injection systems did not differ significantly between the two dosing regimens. This is the first report on the patient satisfaction with these two systems. A recent study by Gold et al. also reported similar findings with respect to patient satisfaction with abaloparatide [18]. Satisfaction with treatment also did not differ significantly between the two injection systems.

Patient satisfaction with the utility of the self-injection system was evaluated using a 12-item questionnaire. The scores in 9 out of 12 items were significantly higher in the 2/W-TPTD group than those in 1/D-TPTD group in the early phase of treatment. Although the trend persisted, the number of items with significant difference gradually decreased as treatment continued and the difference almost disappeared at 26 weeks except for the Q4 (Have you failed to inject the medication?). Those differences may have resulted from the differences between the two injection systems, in particular, in the volume and pH of the injection solution, time and effort of changing the needle, and injection frequency. At the beginning, the high frequency of injection and preparation may have had a greater impact on patients in the 1/D-TPTD group, resulting in a lower satisfaction score, but as the study progressed the patients gradually got used to the routine and scores in both groups appeared to reach similar levels. As for pain at injection, the difference in scores may have been due to a difference in solution pH, 4.4–5.3 for the 2/W-TPTD group and 3.8–4.5 for the 1/D-TPTD group.

Regarding the items for which there were significant differences in patient satisfaction with utility, it is possible that giving patients an adequate explanation of the characteristics of each injection device before administration may lead to appropriate drug selection.

The persistence rate at 26 weeks was 79.4% in the 1/D-TPTD group and 72.6% in the 2/W-TPTD group and was slightly higher than the rates in previous reports evaluating persistence of TPTDs that used real-world data from the Japanese population [12, 19]. The seemingly higher rates may be due to follow-up by the investigators in which patients were asked about how well they were coping with self-injection and explaining to patients the importance of medication adherence, which may have motivated patients to continue self-injection. If this is the case, then the same strategy should be used in real-world clinical practice. The recent report showing high adherence to abaloparatide with high satisfaction from a real-world experience perspective [18] is encouraging, with the authors noting that 55% of patients discussed treatment options in detail with their healthcare team and 27% frequently asked their healthcare team questions regarding treatment choices [18]. Similar results indicating the importance of patient education for adherence to and persistence with teriparatide therapy have been also reported [20, 21].

New clinical fracture occurred in 4 patients (2.2%) in the 2/W-TPTD group. However, these fractures were detected within 2 months after the start of the study and possibly therefore unrelated to the treatment. No statistically significant difference between the groups was observed in this regard.

Utility (EQ-5D) score and pain VAS score analysis indicates significant improvement at 26 weeks, similar to previous reports [22, 23].

### Limitation

The data presented here are from a randomized, allocation-controlled study and may differ from the data obtained in actual clinical situations. However, it is interesting to note that there are some differences between the two dosing regimens (two TPTD self-injection devices). The present results were based on information obtained in the first quarter of the 2-year study period. It is hoped that the post-crossover results after the first quartile study will make the differences between the two dosing regimens with TPTD self-injection devices more compelling.

### Conclusion

The present study demonstrated that overall satisfaction and effectiveness of treatment were similar between the 1/D-TPTD and 2/W-TPTD groups. The finding of a between-group difference in satisfaction with the utility of the self-injection device was useful information for the treatment of osteoporosis in real-world clinical practice. Both medication regimens were well tolerated without serious adverse events.

## Supplementary Information

Below is the link to the electronic supplementary material.

Supplementary file1 (DOCX 28 KB)

Supplementary file2 (DOCX 51 KB)

## Data Availability

The data that support the findings of this study are available from the corresponding author upon reasonable request.
