# Supplementary material for: A crossover comparison of patient satisfaction with two teriparatide regimens: primary results of the Japanese Osteoporosis Intervention Trial 06 (JOINT-06)
Source: J Bone Miner Metab. 2024 Jun 11;42(5):582–90. doi: 10.1007/s00774-024-01521-7 (PMC11455704; doi:10.1007/s00774-024-01521-7)
Supplement: Supplementary file 1 — Supplementary file1 (DOCX 28 KB) [file 774_2024_1521_MOESM1_ESM.docx]

**A crossover comparison of patient satisfaction with two teriparatide regimens. Primary results of the Japanese Osteoporosis Intervention Trial 06 (JOINT-06)**

*Journal of Bone and Mineral Metabolism*

Satoshi Soen^1^, Yukari Uemura^2^, Shiro Tanaka^3^, Yasuhiro Takeuchi^4^, Naoto Endo^5^, Junichi Takada^6^, Satoshi Ikeda^7^, Jun Iwamoto^8^, Nobukazu Okimoto^9^, Sakae Tanaka^10^

^1^Soen Orthopaedics, Osteoporosis and Rheumatology Clinic, Kobe, Hyogo, Japan

^2^Biostatistics Section, Department of Data Science, Center for Clinical Sciences, National Center for Global Health and Medicine, Tokyo, Japan

^3^Department of Clinical Biostatistics, Graduate School of Medicine, Kyoto University, Kyoto, Japan

^4^Toranomon Hospital Endocrine Center, Minato-Ku, Tokyo, Japan; Okinaka Memorial Medical Research, Minato-ku, Tokyo, Japan

^5^ Department of Orthopedic Surgery, Saiseikai Niigata Kenoh Kikan Hospital, Sanjo City, Niigata, Japan

^6^Osteoporosis Center, Sapporo Maruyama Orthopaedic Hospital, Sapporo, Hokkaido, Japan

^7^Department of Orthopaedic Surgery, Ken-Ai Memorial Hospital, Onga, Fukuoka, Japan

^8^Bone and Joint Disease Center, Keiyu Orthopaedic Hospital, Gunma, Japan

^9^Okimoto Clinic, Kure, Hiroshima, Japan

^10^Department of Orthopaedic Surgery, Faculty of Medicine, The University of Tokyo, Tokyo, Japan

**Corresponding author:** Satoshi Soen

Soen Orthopaedics, Osteoporosis and Rheumatology Clinic, Kobe, Hyogo, Japan

Tel: +81-78-413-0088

E-mail: nra48207@nifty.com

Online Resource 1 Patient satisfaction questionnaire

| Category |  | Questions | Answer |
| --- | --- | --- | --- |
| Overall satisfaction |  | How would you rate your satisfaction with this teriparatide preparation? Please rate your satisfaction on a scale of 0 to 5. | (0: dissatisfied - 5: satisfied) |
| Effectiveness of treatment | Q1 | On the teriparatide preparation in use, do you feel positive effects? Please rate the effects on a scale of 0 to 5. | (0: no - 5: yes) |
|  | Q2 | On the teriparatide preparation in use, do you feel improvement of lower or upper back pain? Please rate it on a scale of 0 to 5. | (0: no - 5: yes) |
| Utility of self-injection device | Q1 | How do you feel about the preparation for injection? Please evaluate on a scale of 0 to 5. | (0: difficult - 5: easy) |
|  | Q2 | During injection, how do you feel about the handling and usability of this injection device? | (0: difficult - 5: easy) |
|  | Q3 | After injection, can you confirm whether your injection was successful? Please rate the process on a scale of 0 to 5. | (0: difficult - 5: easy) |
|  | Q4 | Have you experienced failed injection? Please specify how often you failed during the past 2 weeks or 1 month by placing a check in the appropriate checkbox. | (0: none; 4: ≥ 4 times; 9: unknown) |
|  | Q5 | How long does it take for you to complete the whole injection procedure, from preparation to finish? Please specify it by placing a check mark in the appropriate checkbox. | (1: 0-5 min; 2: 5-10 min; 3: ≥10 min) |
|  | Q6 | How do you feel about the frequency of injection? Please rate it on a scale 0 to 5. | (0: dissatisfied - 5: satisfied) |
|  | Q7 | How do you feel about continuing self-injection? Please rate it on a scale of 0 to 5. | (0: difficult - 5: easy) |
|  | Q8 | How do you feel about the safety of self-injection? Please rate it on a scale of 0 to 5. | (0: unsafe - 5: safe) |
|  | Q9 | Do you have pain at the injection site? Please rate it on a scale of 0 to 5. | (0: yes - 5: no) |
|  | Q10 | How do you feel about the storage of the injection set (syringe, needle, etc.)? Please rate your feeling on a scale of 0 to 5. | (0: difficult - 5: easy) |
|  | Q11 | How do you feel about the storage and disposal of used injection sets? Please rate your feeling on a scale of 0 to 5. | (0: difficult - 5: easy) |
|  | Q12 | How do you feel about self-injection? Please rate your feeling on a scale of 0 to 5. | (0: difficult - 5: easy) |
