# Supplementary material for: A crossover comparison of patient satisfaction with two teriparatide regimens: primary results of the Japanese Osteoporosis Intervention Trial 06 (JOINT-06)
Source: J Bone Miner Metab. 2024 Jun 11;42(5):582–90. doi: 10.1007/s00774-024-01521-7 (PMC11455704; doi:10.1007/s00774-024-01521-7)
Supplement: Supplementary file 2 — Supplementary file2 (DOCX 51 KB) [file 774_2024_1521_MOESM2_ESM.docx]

**A crossover comparison of patient satisfaction with two teriparatide regimens. Primary results of the Japanese Osteoporosis Intervention Trial 06 (JOINT-06)**

*Journal of Bone and Mineral Metabolism*

Satoshi Soen^1^, Yukari Uemura^2^, Shiro Tanaka^3^, Yasuhiro Takeuchi^4^, Naoto Endo^5^, Junichi Takada^6^, Satoshi Ikeda^7^, Jun Iwamoto^8^, Nobukazu Okimoto^9^, Sakae Tanaka^10^

^1^Soen Orthopaedics, Osteoporosis and Rheumatology Clinic, Kobe, Hyogo, Japan

^2^Biostatistics Section, Department of Data Science, Center for Clinical Sciences, National Center for Global Health and Medicine, Tokyo, Japan

^3^Department of Clinical Biostatistics, Graduate School of Medicine, Kyoto University, Kyoto, Japan

^4^Toranomon Hospital Endocrine Center, Minato-Ku, Tokyo, Japan; Okinaka Memorial Medical Research, Minato-ku, Tokyo, Japan

^5^ Department of Orthopedic Surgery, Saiseikai Niigata Kenoh Kikan Hospital, Sanjo City, Niigata, Japan

^6^Osteoporosis Center, Sapporo Maruyama Orthopaedic Hospital, Sapporo, Hokkaido, Japan

^7^Department of Orthopaedic Surgery, Ken-Ai Memorial Hospital, Onga, Fukuoka, Japan

^8^Bone and Joint Disease Center, Keiyu Orthopaedic Hospital, Gunma, Japan

^9^Okimoto Clinic, Kure, Hiroshima, Japan

^10^Department of Orthopaedic Surgery, Faculty of Medicine, The University of Tokyo, Tokyo, Japan

**Corresponding author:** Satoshi Soen

Soen Orthopaedics, Osteoporosis and Rheumatology Clinic, Kobe, Hyogo, Japan

Tel: +81-78-413-0088

E-mail: nra48207@nifty.com

Online Resource 2 Patient-reported satisfaction with the self-injection device utility

|  |  |  |  | Response (N, %) | | | | | | |
| --- | --- | --- | --- | --- | --- | --- | --- | --- | --- | --- |
| Time point |  | Questions |  | 0 | 1 | 2 | 3 | 4 | 5 | p |
| After 2 weeks treatment | Q1 | How do you feel about the preparation for injection? | 1/D-TPTD | 2 (1.1%) | 10 (5.6%) | 10 (5.6%) | 36 (20.0%) | 45 (25.0%) | 63 (35.0%) | 0.015 |
|  |  |  | 2/W-TPTD | 2 (1.1%) | 6 (3.4%) | 6 (3.4%) | 28 (15.6%) | 30 (16.8%) | 89 (49.7%) |  |
|  | Q2 | During injection, how do you feel about the handling and usability of this injection device? | 1/D-TPTD | 2 (1.1%) | 10 (5.6%) | 20 (11.1%) | 33 (18.3%) | 47 (26.1%) | 54 (30.0%) | 0.006 |
|  |  |  | 2/W-TPTD | 2 (1.1%) | 3 (1.7%) | 15 (8.4%) | 26 (14.5%) | 36 (20.1%) | 79 (44.1%) |  |
|  | Q3 | After injection, can you confirm whether your injection was successful? | 1/D-TPTD | 4 (2.2%) | 7 (3.9%) | 9 (5.0%) | 46 (25.6%) | 39 (21.7%) | 61 (33.9%) | 0.001 |
|  |  |  | 2/W-TPTD | 1 (0.6%) | 3 (1.7%) | 10 (5.6%) | 27 (15.1%) | 28 (15.6%) | 92 (51.4%) |  |
|  | Q4 | Have you experienced failed injection? Please specify how often you failed during the past 2 weeks or 1 month by placing a check mark in the appropriate checkbox. | 1/D-TPTD | 129 (71.7%) | 24 (13.3%) | 4 (2.2%) | 2 (1.1%) | 4 (2.2%) | 3 (1.7%) | 0.061 |
|  |  |  | 2/W-TPTD | 139 (77.7%) | 13 (7.3%) | 3 (1.7%) | 2 (1.1%) | 1 (0.6%) | 3 (1.7%) |  |
|  | Q5 | How long does it take for you to complete the whole injection procedure, from preparation to finish? | 1/D-TPTD | 0 | 113 (62.8%) | 47 (26.1%) | 6 (3.3%) | - | - | 0.024 |
|  |  |  | 2/W-TPTD | 0 | 95 (53.1%) | 50 (27.9%) | 16 (8.9%) | - | - |  |
|  | Q6 | How do you feel about the frequency of injection? | 1/D-TPTD | 7 (3.9%) | 15 (8.3%) | 16 (8.9%) | 54 (30.0%) | 29 (16.1%) | 45 (25.0%) | < 0.001 |
|  |  |  | 2/W-TPTD | 2 (1.1%) | 6 (3.4%) | 10 (5.6%) | 44 (24.6%) | 26 (14.5%) | 73 (40.8%) |  |
|  | Q7 | How do you feel about continuing self-injection? | 1/D-TPTD | 4 (2.2%) | 10 (5.6%) | 16 (8.9%) | 50 (27.8%) | 35 (19.4%) | 51 (28.3%) | 0.010 |
|  |  |  | 2/W-TPTD | 5 (2.8%) | 5 (2.8%) | 8 (4.5%) | 35 (19.6%) | 37 (20.7%) | 71 (39.7%) |  |
|  | Q8 | How do you feel about the safety of self-injection? | 1/D-TPTD | 2 (1.1%) | 4 (2.2%) | 15 (8.3%) | 42 (23.3%) | 39 (21.7%) | 64 (35.6%) | 0.001 |
|  |  |  | 2/W-TPTD | 1 (0.6%) | 2 (1.1%) | 2 (1.1%) | 35 (19.6%) | 33 (18.4%) | 88 (49.2%) |  |
|  | Q9 | Do you have pain at the injection site? | 1/D-TPTD | 2 (1.1%) | 11 (6.1%) | 11 (6.1%) | 28 (15.6%) | 51 (28.3%) | 63 (35.0%) | 0.002 |
|  |  |  | 2/W-TPTD | 3 (1.7%) | 3 (1.7%) | 10 (5.6%) | 16 (8.9%) | 31 (17.3%) | 98 (54.7%) |  |
|  | Q10 | How do you feel about the storage of the injection set (syringe, needle, etc.)? | 1/D-TPTD | 2 (1.1%) | 4 (2.2%) | 5 (2.8%) | 40 (22.2%) | 35 (19.4%) | 80 (44.4%) | 0.148 |
|  |  |  | 2/W-TPTD | 3 (1.7%) | 3 (1.7%) | 5 (2.8%) | 28 (15.6%) | 24 (13.4%) | 98 (54.7%) |  |
|  | Q11 | How do you feel about the storage and disposal of used injection sets? | 1/D-TPTD | 1 (0.6%) | 4 (2.2%) | 6 (3.3%) | 31 (17.2%) | 44 (24.4%) | 80 (44.4%) | 0.136 |
|  |  |  | 2/W-TPTD | 4 (2.2%) | 2 (1.1%) | 5 (2.8%) | 25 (14.0%) | 18 (10.1%) | 107 (59.8%) |  |
|  | Q12 | How do you feel about self-injection? | 1/D-TPTD | 6 (3.3%) | 9 (5.0%) | 12 (6.7%) | 49 (27.2%) | 40 (22.2%) | 50 (27.8%) | 0.002 |
|  |  |  | 2/W-TPTD | 3 (1.7%) | 7 (3.9%) | 9 (5.0%) | 31 (17.3%) | 29 (16.2%) | 82 (45.8%) |  |
| After 4 weeks treatment | Q1 | How do you feel about the preparation for injection? | 1/D-TPTD | 2 (1.1%) | 3 (1.7%) | 9 (5.0%) | 28 (15.6%) | 32 (17.8%) | 92 (51.1%) | 0.315 |
|  |  |  | 2/W-TPTD | 1 (0.6%) | 3 (1.7%) | 4 (2.2%) | 28 (15.6%) | 27 (15.1%) | 96 (53.6%) |  |
|  | Q2 | During injection, how do you feel about the handling and usability of this injection device? | 1/D-TPTD | 3 (1.7%) | 6 (3.3%) | 9 (5.0%) | 27 (15.0%) | 39 (21.7%) | 82 (45.6%) | 0.072 |
|  |  |  | 2/W-TPTD | 0 | 5 (2.8%) | 6 (3.4%) | 23 (12.8%) | 32 (17.9%) | 93 (52.0%) |  |
|  | Q3 | After injection, can you confirm whether your injection was successful? | 1/D-TPTD | 3 (1.7%) | 5 (2.8%) | 8 (4.4%) | 26 (14.4%) | 38 (21.1%) | 86 (47.8%) | 0.022 |
|  |  |  | 2/W-TPTD | 1 (0.6%) | 2 (1.1%) | 6 (3.4%) | 20 (11.2%) | 27 (15.1%) | 103 (57.5%) |  |
|  | Q4 | Have you experienced failed injection? Please specify how often you failed during the past 2 weeks or 1 month by placing a check mark in the appropriate checkbox. | 1/D-TPTD | 122 (67.8%) | 25 (13.9%) | 7 (3.9%) | 6 (3.3%) | 2 (1.1%) | 4 (2.2%) | 0.001 |
|  |  |  | 2/W-TPTD | 146 (81.6%) | 7 (3.9%) | 4 (2.2%) | 1 (0.6%) | 1 (0.6%) | 0 |  |
|  | Q5 | How long does it take for you to complete the whole injection procedure, from preparation to finish? | 1/D-TPTD | 0 | 116 (64.4%) | 45 (25.0%) | 5 (2.8%) | - | - | 0.130 |
|  |  |  | 2/W-TPTD | 0 | 101 (56.4%) | 48 (26.8%) | 10 (5.6%) | - | - |  |
|  | Q6 | How do you feel about the frequency of injection? | 1/D-TPTD | 3 (1.7%) | 12 (6.7%) | 17 (9.4%) | 41 (22.8%) | 31 (17.2%) | 62 (34.4%) | 0.021 |
|  |  |  | 2/W-TPTD | 1 (0.6%) | 3 (1.7%) | 6 (3.4%) | 49 (27.4%) | 34 (19.0%) | 66 (36.9%) |  |
|  | Q7 | How do you feel about continuing self-injection? | 1/D-TPTD | 3 (1.7%) | 9 (5.0%) | 16 (8.9%) | 35 (19.4%) | 28 (15.6%) | 75 (41.7%) | 0.071 |
|  |  |  | 2/W-TPTD | 2 (1.1%) | 5 (2.8%) | 9 (5.0%) | 30 (16.8%) | 31 (17.3%) | 82 (45.8%) |  |
|  | Q8 | How do you feel about the safety of self-injection? | 1/D-TPTD | 2 (1.1%) | 3 (1.7%) | 10 (5.6%) | 35 (19.4%) | 31 (17.2%) | 85 (47.2%) | 0.015 |
|  |  |  | 2/W-TPTD | 0 | 0 | 4 (2.2%) | 31 (17.3%) | 28 (15.6%) | 96 (53.6%) |  |
|  | Q9 | Do you have pain at the injection site? | 1/D-TPTD | 3 (1.7%) | 12 (6.7%) | 11 (6.1%) | 23 (12.8%) | 49 (27.2%) | 68 (37.8%) | 0.004 |
|  |  |  | 2/W-TPTD | 1 (0.6%) | 5 (2.8%) | 7 (3.9%) | 16 (8.9%) | 43 (24.0%) | 87 (48.6%) |  |
|  | Q10 | How do you feel about the storage of the injection set (syringe, needle, etc.)? | 1/D-TPTD | 4 (2.2%) | 1 (0.6%) | 3 (1.7%) | 28 (15.6%) | 31 (17.2%) | 99 (55.0%) | 0.338 |
|  |  |  | 2/W-TPTD | 0 | 1 (0.6%) | 4 (2.2%) | 26 (14.5%) | 30 (16.8%) | 98 (54.7%) |  |
|  | Q11 | How do you feel about the storage and disposal of used injection sets? | 1/D-TPTD | 4 (2.2%) | 1 (0.6%) | 7 (3.9%) | 24 (13.3%) | 28 (15.6%) | 102 (56.7%) | 0.097 |
|  |  |  | 2/W-TPTD | 1 (0.6%) | 0 | 3 (1.7%) | 21 (11.7%) | 30 (16.8%) | 104 (58.1%) |  |
|  | Q12 | How do you feel about self-injection? | 1/D-TPTD | 6 (3.3%) | 6 (3.3%) | 9 (5.0%) | 36 (20.0%) | 36 (20.0%) | 73 (40.6%) | 0.014 |
|  |  |  | 2/W-TPTD | 1 (0.6%) | 1 (0.6%) | 7 (3.9%) | 34 (19.0%) | 31 (17.3%) | 85 (47.5%) |  |
| After 13 weeks treatment | Q1 | How do you feel about the preparation for injection? | 1/D-TPTD | 0 | 1 (0.6%) | 4 (2.2%) | 20 (11.1%) | 32 (17.8%) | 101 (56.1%) | 0.277 |
|  |  |  | 2/W-TPTD | 1 (0.6%) | 0 | 3 (1.7%) | 12 (6.7%) | 28 (15.6%) | 102 (57.0%) |  |
|  | Q2 | During injection, how do you feel about the handling and usability of this injection device? | 1/D-TPTD | 0 | 1 (0.6%) | 3 (1.7%) | 26 (14.4%) | 36 (20.0%) | 92 (51.1%) | 0.243 |
|  |  |  | 2/W-TPTD | 1 (0.6%) | 1 (0.6%) | 3 (1.7%) | 16 (8.9%) | 26 (14.5%) | 99 (55.3%) |  |
|  | Q3 | After injection, can you confirm whether your injection was successful? | 1/D-TPTD | 0 | 1 (0.6%) | 4 (2.2%) | 24 (13.3%) | 34 (18.9%) | 95 (52.8%) | 0.048 |
|  |  |  | 2/W-TPTD | 1 (0.6%) | 0 | 4 (2.2%) | 11 (6.1%) | 23 (12.8%) | 107 (59.8%) |  |
|  | Q4 | Have you experienced failed injection? Please specify how often you failed during the past 2 weeks or 1 month by placing a check mark in the appropriate checkbox. | 1/D-TPTD | 138 (76.7%) | 16 (8.9%) | 1 (0.6%) | 2 (1.1%) | 0 | 1 (0.6%) | 0.115 |
|  |  |  | 2/W-TPTD | 136 (76.0%) | 6 (3.4%) | 1 (0.6%) | 1 (0.6%) | 0 | 2 (1.1%) |  |
|  | Q5 | How long does it take for you to complete the whole injection procedure, from preparation to finish? | 1/D-TPTD | 0 | 133 (73.9%) | 24 (13.3%) | 1 (0.6%) | - | - | 0.001 |
|  |  |  | 2/W-TPTD | 0 | 101 (56.4%) | 36 (20.1%) | 9 (5.0%) | - | - |  |
|  | Q6 | How do you feel about the frequency of injection? | 1/D-TPTD | 1 (0.6%) | 9 (5.0%) | 15 (8.3%) | 40 (22.2%) | 24 (13.3%) | 69 (38.3%) | 0.119 |
|  |  |  | 2/W-TPTD | 1 (0.6%) | 2 (1.1%) | 11 (6.1%) | 33 (18.4%) | 32 (17.9%) | 67 (37.4%) |  |
|  | Q7 | How do you feel about continuing self-injection? | 1/D-TPTD | 0 | 7 (3.9%) | 10 (5.6%) | 39 (21.7%) | 19 (10.6%) | 83 (46.1%) | 0.795 |
|  |  |  | 2/W-TPTD | 2 (1.1%) | 3 (1.7%) | 14 (7.8%) | 22 (12.3%) | 30 (16.8%) | 75 (41.9%) |  |
|  | Q8 | How do you feel about the safety of self-injection? | 1/D-TPTD | 1 (0.6%) | 3 (1.7%) | 5 (2.8%) | 27 (15.0%) | 31 (17.2%) | 91 (50.6%) | 0.390 |
|  |  |  | 2/W-TPTD | 2 (1.1%) | 2 (1.1%) | 5 (2.8%) | 17 (9.5%) | 26 (14.5%) | 94 (52.5%) |  |
|  | Q9 | Do you have pain at the injection site? | 1/D-TPTD | 3 (1.7%) | 12 (6.7%) | 21 (11.7%) | 29 (16.1%) | 40 (22.2%) | 53 (29.4%) | < 0.001 |
|  |  |  | 2/W-TPTD | 3 (1.7%) | 6 (3.4%) | 4 (2.2%) | 14 (7.8%) | 38 (21.2%) | 81 (45.3%) |  |
|  | Q10 | How do you feel about the storage of the injection set (syringe, needle, etc.)? | 1/D-TPTD | 0 | 2 (1.1%) | 1 (0.6%) | 23 (12.8%) | 27 (15.0%) | 105 (58.3%) | 0.697 |
|  |  |  | 2/W-TPTD | 1 (0.6%) | 1 (0.6%) | 2 (1.1%) | 14 (7.8%) | 29 (16.2%) | 99 (55.3%) |  |
|  | Q11 | How do you feel about the storage and disposal of used injection sets? | 1/D-TPTD | 0 | 1 (0.6%) | 2 (1.1%) | 26 (14.4%) | 22 (12.2%) | 107 (59.4%) | 0.915 |
|  |  |  | 2/W-TPTD | 2 (1.1%) | 1 (0.6%) | 2 (1.1%) | 17 (9.5%) | 22 (12.3%) | 102 (57.0%) |  |
|  | Q12 | How do you feel about self-injection? | 1/D-TPTD | 0 | 7 (3.9%) | 4 (2.2%) | 28 (15.6%) | 33 (18.3%) | 86 (47.8%) | 0.532 |
|  |  |  | 2/W-TPTD | 0 | 1 (0.6%) | 14 (7.8%) | 17 (9.5%) | 28 (15.6%) | 86 (48.0%) |  |
| After 26 weeks treatment | Q1 | How do you feel about the preparation for injection? | 1/D-TPTD | 5 (2.8%) | 1 (0.6%) | 3 (1.7%) | 12 (6.7%) | 29 (16.1%) | 99 (55.0%) | 0.633 |
|  |  |  | 2/W-TPTD | 1 (0.6%) | 1 (0.6%) | 3 (1.7%) | 19 (10.6%) | 19 (10.6%) | 93 (52.0%) |  |
|  | Q2 | During injection, how do you feel about the handling and usability of this injection device? | 1/D-TPTD | 5 (2.8%) | 1 (0.6%) | 2 (1.1%) | 14 (7.8%) | 27 (15.0%) | 100 (55.6%) | 0.853 |
|  |  |  | 2/W-TPTD | 1 (0.6%) | 1 (0.6%) | 3 (1.7%) | 22 (12.3%) | 17 (9.5%) | 92 (51.4%) |  |
|  | Q3 | After injection, can you confirm whether your injection was successful? | 1/D-TPTD | 1 (0.6%) | 4 (2.2%) | 5 (2.8%) | 17 (9.4%) | 26 (14.4%) | 96 (53.3%) | 0.776 |
|  |  |  | 2/W-TPTD | 1 (0.6%) | 0 | 4 (2.2%) | 23 (12.8%) | 20 (11.2%) | 88 (49.2%) |  |
|  | Q4 | Have you experienced failed injection? Please specify how often you failed during the past 2 weeks or 1 month by placing a check mark in the appropriate checkbox. | 1/D-TPTD | 124 (68.9%) | 16 (8.9%) | 4 (2.2%) | 2 (1.1%) | 3 (1.7%) | 0 | 0.005 |
|  |  |  | 2/W-TPTD | 128 (71.5%) | 6 (3.4%) | 0 | 0 | 1 (0.6%) | 1 (0.6%) |  |
|  | Q5 | How long does it take for you to complete the whole injection procedure, from preparation to finish? | 1/D-TPTD | 0 | 122 (67.8%) | 22 (12.2%) | 5 (2.8%) | - | - | 0.207 |
|  |  |  | 2/W-TPTD | 0 | 104 (58.1%) | 24 (13.4%) | 8 (4.5%) | - | - |  |
|  | Q6 | How do you feel about the frequency of injection? | 1/D-TPTD | 6 (3.3%) | 6 (3.3%) | 7 (3.9%) | 37 (20.6%) | 27 (15.0%) | 66 (36.7%) | 0.433 |
|  |  |  | 2/W-TPTD | 2 (1.1%) | 5 (2.8%) | 9 (5.0%) | 35 (19.6%) | 17 (9.5%) | 68 (38.0%) |  |
|  | Q7 | How do you feel about continuing self-injection? | 1/D-TPTD | 7 (3.9%) | 6 (3.3%) | 6 (3.3%) | 24 (13.3%) | 25 (13.9%) | 81 (45.0%) | 0.812 |
|  |  |  | 2/W-TPTD | 2 (1.1%) | 4 (2.2%) | 9 (5.0%) | 33 (18.4%) | 23 (12.8%) | 65 (36.3%) |  |
|  | Q8 | How do you feel about the safety of self-injection? | 1/D-TPTD | 2 (1.1%) | 2 (1.1%) | 2 (1.1%) | 19 (10.6%) | 28 (15.6%) | 96 (53.3%) | 0.407 |
|  |  |  | 2/W-TPTD | 0 | 0 | 2 (1.1%) | 19 (10.6%) | 26 (14.5%) | 89 (49.7%) |  |
|  | Q9 | Do you have pain at the injection site? | 1/D-TPTD | 0 | 10 (5.6%) | 17 (9.4%) | 27 (15.0%) | 41 (22.8%) | 54 (30.0%) | 0.146 |
|  |  |  | 2/W-TPTD | 2 (1.1%) | 5 (2.8%) | 9 (5.0%) | 23 (12.8%) | 38 (21.2%) | 59 (33.0%) |  |
|  | Q10 | How do you feel about the storage of the injection set (syringe, needle, etc.)? | 1/D-TPTD | 0 | 1 (0.6%) | 2 (1.1%) | 13 (7.2%) | 28 (15.6%) | 105 (58.3%) | 0.283 |
|  |  |  | 2/W-TPTD | 0 | 2 (1.1%) | 3 (1.7%) | 19 (10.6%) | 18 (10.1%) | 94 (52.5%) |  |
|  | Q11 | How do you feel about the storage and disposal of used injection sets? | 1/D-TPTD | 0 | 2 (1.1%) | 5 (2.8%) | 13 (7.2%) | 23 (12.8%) | 106 (58.9%) | 0.673 |
|  |  |  | 2/W-TPTD | 0 | 1 (0.6%) | 0 | 19 (10.6%) | 18 (10.1%) | 98 (54.7%) |  |
|  | Q12 | How do you feel about self-injection? | 1/D-TPTD | 3 (1.7%) | 7 (3.9%) | 2 (1.1%) | 24 (13.3%) | 22 (12.2%) | 91 (50.6%) | 0.743 |
|  |  |  | 2/W-TPTD | 1 (0.6%) | 5 (2.8%) | 6 (3.4%) | 26 (14.5%) | 20 (11.2%) | 78 (43.6%) |  |

Values are indicated as number and percentage. Differences between the groups were tested by using the Cochran-Mantel-Haenszel-test.

1/D-TPTD, once-daily dose of teriparatide; 2/W-TPTD, twice-weekly dose of teriparatide
